# Influence of Scanning Speed on the Microstructure and Wear Resistance of Laser Alloying Coatings on Ti-6Al-4V Substrate

**DOI:** 10.3390/ma15175819

**Published:** 2022-08-24

**Authors:** Huijun Yu, Xiaoxi Meng, Zifan Wang, Chuanzhong Chen

**Affiliations:** 1 Key Laboratory of High Efficiency and Clean Mechanical Manufacture, Ministry of Education, and National Demonstration Center for Experimental Mechanical Engineering Education, School of Mechanical Engineering, Shandong University, Jinan 250061, China; 2 Key Laboratory for Liquid-Solid Structural Evolution and Processing of Materials, Ministry of Education, and Shandong Engineering & Technology Research Center for Superhard Material, School of Materials Science and Engineering, Shandong University, Jinan 250061, China

**Keywords:** laser alloying, titanium alloy, scanning speed, microstructure, wear resistance

## Abstract

Laser alloying has attracted significant attentions due to the advantages of high processing precision, good controllability and low heat effects on the substrate. However, the complexity of laser alloying requires further attentions on its processing parameters. This study aims at improving the wear resistance of the Ti-6Al-4V substrate by means of laser surface alloying with Ni-coated graphite (G@Ni). The effect of laser scanning speed is explored. The result suggests that the coating has a high surface quality and excellent metallurgical bonding with the substrate. NiTi and NiTi_2_ have a eutectic microstructure as well as in the TiC ceramic-reinforced phase as dendrites distribute in the γ-Ni matrix of the coatings. At higher scanning speeds, the lower energy density and shorter existence time of the molten pool refines the microstructure of the coating, improving its microhardness. At the scanning speed of 15 mm/s, the coating has the lowest wear weight loss due to its high microhardness and dense structure. This paper explores the influence of scanning speed on the microstructure and properties of the coatings, expanding the application of laser alloying on the surface modification of Ti-6Al-4V alloys.

## 1. Introduction

Titanium (Ti) and its alloys are widely used in marine, aerospace, automotive, biomedical, power generation, chemical industry, and other fields due to its excellent properties such as having a light weight, good corrosion resistance, high specific strength, bio-compatibility and oxidation resistance [1,2,3]. Among them, Ti-6Al-4V has become one of the most commonly used titanium alloys in the industry for its good comprehensive properties [4]. Ti-6Al-4V has a strength similar to steel, while its density is about 40% lower. It is an α + β alloy containing both α and β stabilizers (aluminum and vanadium) [5,6,7], which have a better corrosion resistance, a lower weight, and a higher strength [8,9].

With the development of industrial technology, the demand for titanium and its alloys further increases. However, low hardness and poor wear resistance severely limit their applications as transmission components [10,11]. In order to improve the properties of titanium alloys, a range of surface modification methods have been employed, which mainly include chemical heat treatment [12,13], ion implantation [14,15], micro-arc oxidation [16,17], electroplating [18], vapor deposition [19,20] and thermal spraying [21,22].

With the rapid development of laser technologies, laser surface modification technology has received extensive attentions, providing a new and more effective method for material surface modification. Laser surface modification, which mainly include laser cladding [23,24], laser alloying [25,26,27], laser melting [28,29], and pulsed laser deposition [30,31], is more suitable for the surface modification of metal substrates for the advantages of flexibility, a low process cost, and simple operation. Laser alloying involves the use of a high energy laser to scan and heat the pre-paved modification materials and the surface of the substrate. During the laser alloying progress, the pre-paved materials, commonly including C [32], B [33], Al [25], G@Ni [27], B_4_C [34] and BN [35], melt and solidify rapidly, while reacting with the substrate sufficiently to form ceramic-reinforced phases and intermetallic compounds. The obtained coatings can thus improve the surface hardness and wear resistance of the substrate.

Laser alloying is a complex process of surface modification. Energy density, which is closely related to the laser scanning speed, has a great influence on the macroscopic quality and the property of the produced coatings. In this study, the effects of laser scanning speed on the wear resistance of the coatings on Ti-6Al-4V are studied by examining the macroscopic quality of the coating and analyzing the phase composition and microstructure. By adjusting the laser scanning speed, coatings with excellent metallurgical bonding and improved wear resistance are demonstrated.

## 2. Experimental Materials and Methods

### 2.1. Experimental Materials

This study used Ti-6Al-4V as the substrate, which is a typical α + β phase titanium alloy with outstanding physical and mechanical properties. The chemical composition of Ti-6Al-4V can be seen in Table 1. A Ti-6Al-4V sheet was cut into small pieces of 30 mm × 40 mm × 10 mm in size for characterizations such as XRD and SEM, and 40 mm × 40 mm × 10 mm in size for wear test using a wire-cutting machine. Before powder presetting, the samples were abraded by 180# water sandpapers to remove oxidized films for flat and smooth surfaces and cleaned using absolute ethanol in an ultrasonic cleaner to remove surface attachments.

Ni-coated graphite (G@Ni), a composite powder with graphite as the core and Ni as the coating with a chemical composition of 75 wt.% Ni + 25 wt.% graphite and a particle size ranging 25~45 μm, was used as the laser alloying powder. The G@Ni powder was mixed with binder (Na_2_SiO_3_:H_2_O = 1:3) first and paved onto the Ti-6Al-4V samples. The thickness of the pre-paved coating was controlled to be 1 mm. 

### 2.2. Experimental Methods

A YLS-4000 fiber laser was used in this study for laser alloying, and argon was used to protect the molten pool from oxidation. The gas flow was 10~15 L/min, and the selected laser process parameters were a laser power of 1400 W, a spot diameter of 4.0 mm and a lapping rate of 30%, which were settled according to preliminary experiments. A range of laser scanning speeds of 12 mm/s, 15 mm/s and 18 mm/s were chosen to explore their influence on the microstructures and properties of the coating.

After laser alloying, the samples were cut along a cross-section in the vertical scanning direction into pieces of 10 mm × 10 mm × 10 mm in size, then ground by water sandpapers to obtain a smooth and flat surface. Then, the samples were polished with the assistance of a Cr_2_O_3_ suspension, whereafter, the samples were etched for 10~20 s with a mixed solution of HF and HNO_3_ (*v*/*v* = 1:3). The samples were rinsed and dried as soon as the polished surface turned brown.

The phase composition was analyzed using a Rigaku Ultima IV X-ray diffractometer, with a voltage of 40 kV, a current of 40 mA, a scanning range of 10~90° and a scanning speed of 2°/min. The microstructure was observed using a Hitachi JSM-7800F scanning electron microscope (SEM). The Gibbs free energy of the reactions was calculated according to the classic Gibbs free energy function method, i.e., ΔG = ΔH − T·ΔS. The values of H and S are taken from the study referenced in [36].

The microhardness along the depth of the coating was tested using a DHV-1000 Vickers micro-hardness tester, with a load of 200 g and a loading time of 10 s. During the test, the microhardness of the coating and the substrate were measured, respectively, at 0.1 mm in a perpendicular direction. Three points, each of which had a distance of 0.05 mm from each other in the horizontal direction, were chosen to be tested. The average microhardness of these points was counted as the microhardness of the certain depth.

Wear resistance of the coating and substrate was tested using a HT-1000 wear tester, with a load of 2000 g and a rotational speed of 560 r/min for 30 min. Before the test, the samples were ground to weaken the influence of surface roughness on the wear resistance. After grinding, the samples were rinsed, dried and weighed using a Satorious QuinTix electronic balance with a resolution of 0.1 mg. During the wear test, a Si_3_N_4_ ceramic grinding ball with a microhardness of 1600 HV was used as the abrasive material. The wear surface morphologies of the coating and the substrate were observed using SEM; while the components of the wear debris were analyzed using the attached XMax-80 energy dispersive spectrometer.

## 3. Results and Discussion

### 3.1. Macroscopic Quality of the Coatings

**Figure 1:**
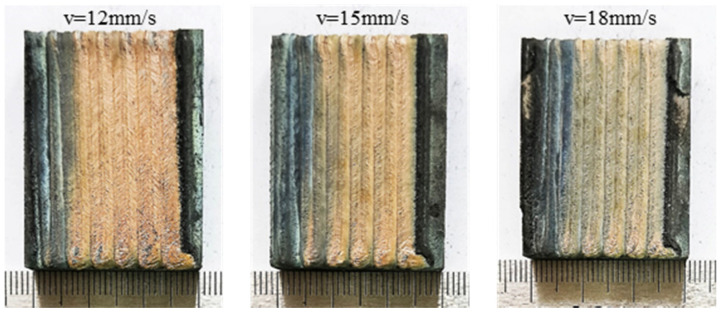
Surface morphologies of the coating under different laser scanning speeds.

Surface morphologies of the laser alloying coatings at different scanning speeds (12 mm/s, 15 mm/s and 18 mm/s) are shown in Figure 1. The quality of the coatings is at a high level, and the coating surface is relatively flat and smooth. The increase in the scanning speed shortened the residence time of the laser beam on the material surface, which decreased the depth and width of the molten pool. Due to the low energy density, the coating could not be completely melted with the substrate to form a molten pool, therefore a small number of un-melted particles appeared at the edges of the melting channel.

**Figure 2:**
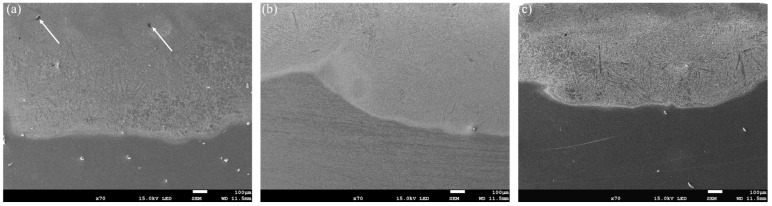
Cross-section morphologies of the coating under different laser scanning speeds: (**a**) ν = 12 mm/s, (**b**) ν = 15 mm/s and (**c**) ν = 18 mm/s.

The cross-sectional, low-magnification morphologies of the coatings at different scanning speeds are shown in Figure 2. The specimens can be divided into three regions, namely the melted zone, the heat-affected zone and the substrate. The melt zone can be further divided into the upper, the medium, the lower and the bonding zones. The distinct fusion line between the coating and the substrate indicates the formation of good metallurgical bonding. The crescent shape of the coating is due to the Gaussian distribution of the energy density of the circular spot, with the highest energy density at the center and lowest energy density at the edge. The coating structure is dense with no obvious cracks. The wider molten pool at low scanning speeds is due to the fact that the laser has a longer interaction time with the substrate, and therefore more materials are molten and reacted in the molten pool. With the increase of the scanning speed, the width and depth of the molten pool gradually decreased. It is worth noting that when the scanning speed was 12 mm/s, a small number of pores appeared in the upper zone of the coating (indicated by the arrows in Figure 2a), but when the scanning speed was increased to 15 mm/s (Figure 2b) and 18 mm/s (Figure 2c), no obvious pores could be observed. With a low scanning speed, the molten pool exists for a longer time. With the limited protective effect of argon, the C in G@Ni could be partially oxidized to generate CO_2_ and form pores. In addition, the vaporization of the liquid melt also causes the formation of pores.

### 3.2. Phase Composition

**Figure 3:**
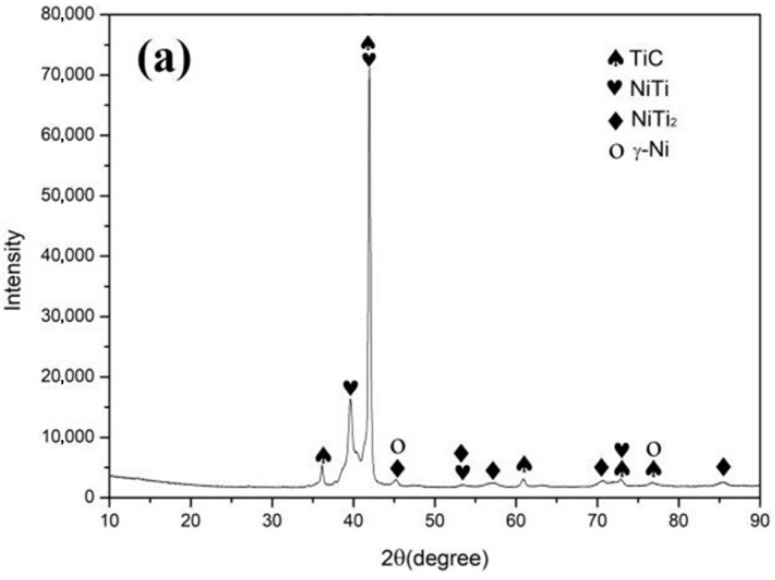

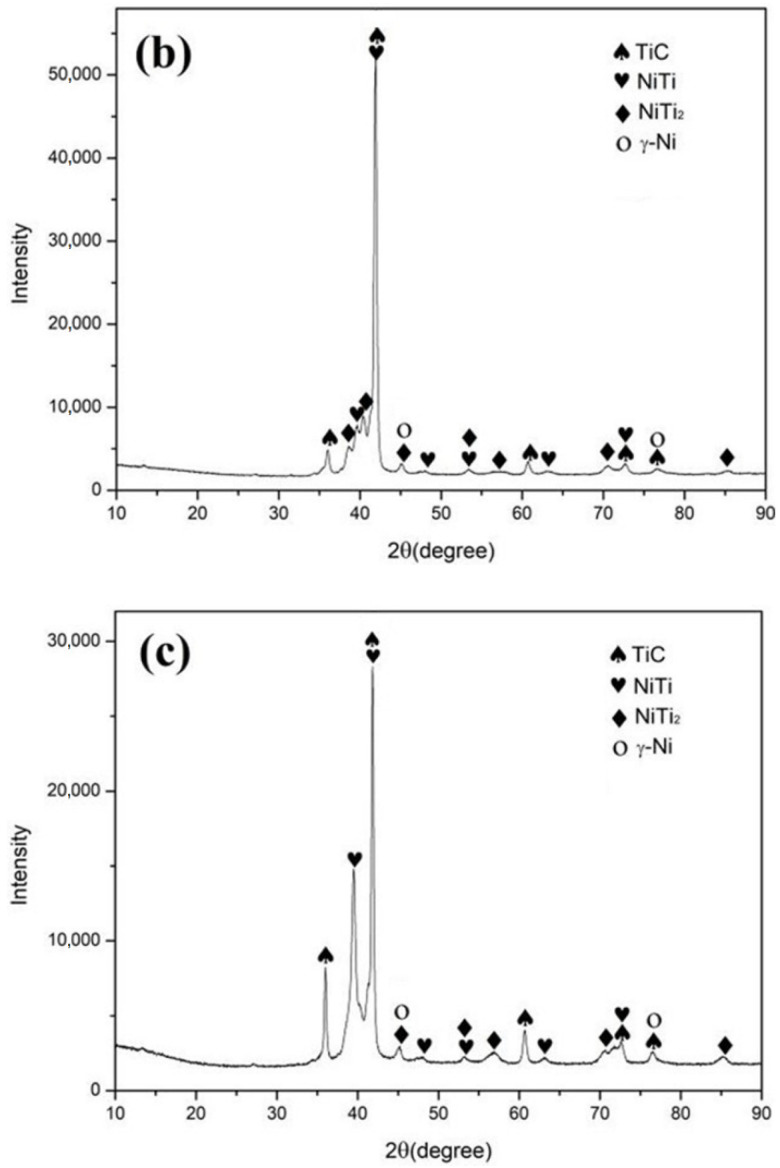
XRD patterns of the coatings: (**a**) ν = 12 mm/s, (**b**) ν = 15 mm/s and (**c**) ν = 18 mm/s.

Figure 3 shows the X-ray diffraction (XRD) patterns of the coating on the surface of the Ti-6Al-4V alloy at different scanning speeds of 12 mm/s (Figure 3a), 15 mm/s (Figure 3b) and 18 mm/s (Figure 3c). The XRD analysis shows that the coating is mainly composed of TiC, NiTi, NiTi_2_ and the solid solution phase, γ-Ni, with a face-centered cubic (fcc) lattice. The characteristic peak of graphite (2θ = 26.5°) is not found in the XRD patterns since graphite was involved in the reaction to form the coatings and was not retained. As can be seen from the patterns, coatings at different scanning speeds have the same compositions of the phases, differing only in the relative content. During the laser alloying process, the surface of the Ti-6Al-4V alloy substrate melted and mixed with the alloying material by the processes of convection and diffusion. The main reactions that took place in the molten pool are as follows:

Ni + Ti → NiTi(1)

Ni + 2Ti → NiTi_2_ (2)

Ti + C → TiC(3)

**Figure 4:**
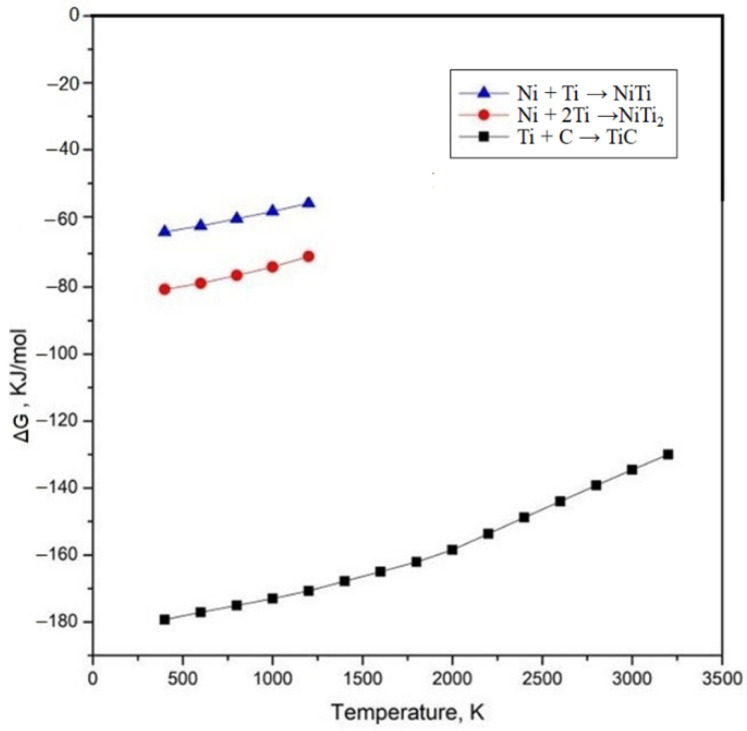
The change of Gibbs free energy of chemical reaction.

According to the curve of the change of standard Gibbs free energy (ΔG) of reactions 1, 2 and 3 with the temperatures shown in Figure 4, the ΔG of reactions 1, 2 and 3 are negative in the temperature range of 400 K to 3200 K, indicating that both chemical reactions can proceed spontaneously in the molten pool. The TiC phase is preferentially precipitated in the molten pool according to the Gibbs free energy theory of reactions, i.e., the smaller the ΔG value, the stronger the thermodynamic driving force of the reaction and the easier it is for it to occur. Since TiC has a NaCl-type face-centered cubic crystal structure, it is easy to form particles or grow into dendrites. Ti and Ni are more likely to form NiTi and NiTi_2_ [37], which is consistent with the XRD results in Figure 3. The matrix of the coating comprises γ-Ni, distributing NiTi, NiTi_2_ and a TiC ceramic-reinforced phase. All three reaction products have a higher hardness compared to the substrate, which is beneficial to improving its hardness and wear resistance. It is clear that TiC is more readily generated during the alloying process.

### 3.3. Microstructure Analysis

**Figure 5:**
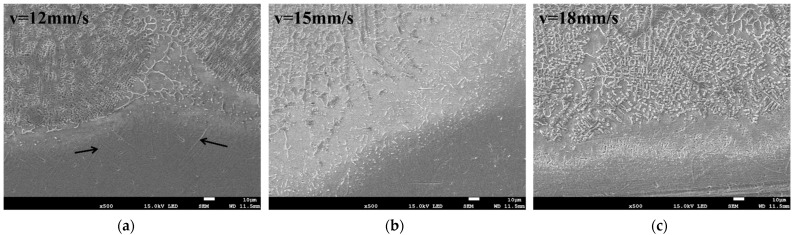
Morphologies of the bonding zone of coatings under different laser scanning speeds.

Figure 5 shows the microstructure of the bonding zone between the coating and the substrate at different scanning speeds. The microstructure of the epitaxial growth could be observed in the bonding zone between the coating and the substrate under higher magnification, further proving that excellent metallurgical bonding occurred. It can be seen that the structure of the bonding zone is mainly solidified in the shape of cellular-dendrites and dendrites and grows roughly in the direction perpendicular to the interface between the coating and the substrate. The microstructure of the bonding zone is closely related to constitutional supercooling at the solid-liquid interface in the molten pool. A greater temperature gradient near the substrate slows the solidification rate with less constitutional supercooling, which can lead to the growth of solidified microstructure in cellular-dendrites. In the solidification process, the solidification rate and the constitutional supercooling increase with the decrease of the temperature gradient of the molten pool is what makes the solidified microstructure gradually change to the form of a dendritic growth. It can be seen from Figure 5 that the substrate presents an obvious heat-affected zone, where the substrate has not melted but the temperature has exceeded the phase change temperature of Ti. Elongated needle-like α phases are observed to be distributed in the original β grains (indicated by the arrows in Figure 5a), which form a net-like microstructure. This is due to the fact that during laser heating, as the temperature of the titanium alloy rises to the β-phase temperature range, the microstructure is transformed from a α + β two-phase to a single β-phase. During the rapid cooling process, the original β phase does not have enough time to diffuse and transform into the equilibrium α phase. Instead, the shear-induced phase transformation is completed by the collective short-range migration of atoms in the β-phase, forming the needle-like martensitic phase α.

**Figure 6:**
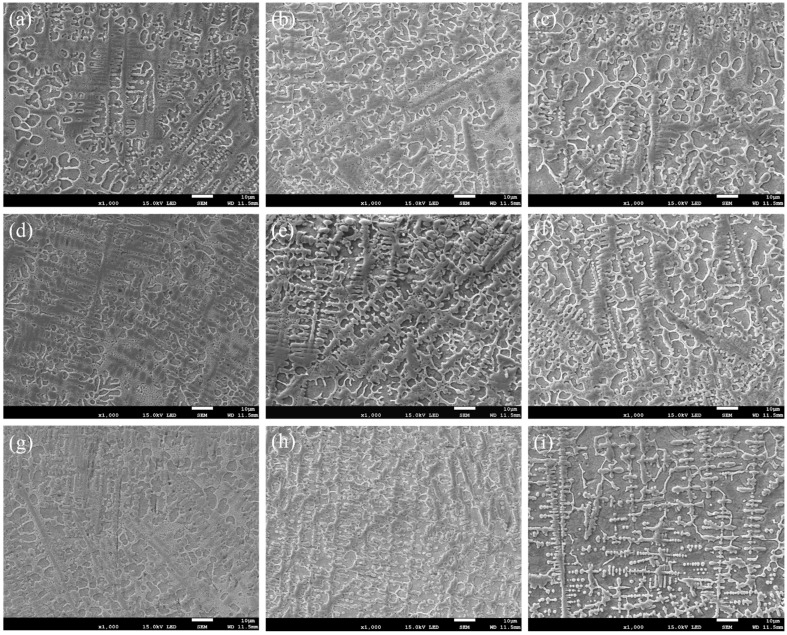
Morphologies of coatings: (**a**–**c**) the upper zone, (**d**–**f**) the medium zone, (**g**–**i**) the lower zone, (**a**,**d**,**g**) v = 12 mm/s, (**b**,**e**,**h**) v = 15 mm/s and (**c**,**f**,**i**) v = 18 mm/s.

The microstructures of different depths of the coating at different scanning speeds are shown in Figure 6. Figure 6a–i show the microstructures of the upper, medium and lower zones of the coating under different scanning speeds, respectively. Dendritic, granular, irregular massive, slate-like and reticulated eutectic morphologies are all observed in the coatings. The TiC phase preferentially precipitates in dendritic formations, followed by the eutectic reaction of the Ni-Ti intermetallic compound to form the eutectic microstructure.

Based on the morphologies of the different zones from the coatings shown in Figure 6, the size of the microstructure shows obvious differences due to the difference in the temperature and the cooling rate in the molten pool. The upper and medium zones possess coarse, irregular, massive microstructures or dendrites (Figure 6a–f), and the lower zones are dominated by granular microstructures (Figure 6g–i). Due to the high laser absorptivity and low thermal conductivity of graphite, the molten pool exists for a relatively long time. The solidification interface gradually advances from the lower zone of the molten pool to the free surface, and the upper zone of the coating melts the first and solidifies the last. The closer it is to the free surface, then the longer the molten pool exists. Therefore, a smaller temperature gradient is expected in the upper zone of the molten pool, which decreases the nucleation rate. The formed primary crystals thus receive more time to grow up, forming a relatively coarse and non-oriented microstructure (Figure 6a–c). At the lower zone of the molten pool, the precipitated grains do not have enough growth time due to the chilling effect of the substrate, leading to finer grain sizes (Figure 6g–i).

From Figure 6, it can be seen that the microstructure of the coating gradually refines from the upper zone, to the medium zone, then to the lower zone as the scanning speed increases, and the microstructure of the same area of the coating from different specimens also gradually refines. When the scanning speed is 12 mm/s, the microstructure of the coating is at its coarsest due to the high laser energy density at the low scanning speed (Figure 6a,d,g). The molten pool exists for a longer time, and the various phases formed fully grow during the solidification process. With the increase of the laser scanning speed, the laser energy density decreases, which shortens the existence duration of the molten pool and also refines the microstructure (Figure 6c,f,i).

### 3.4. Microhardness

**Figure 7:**
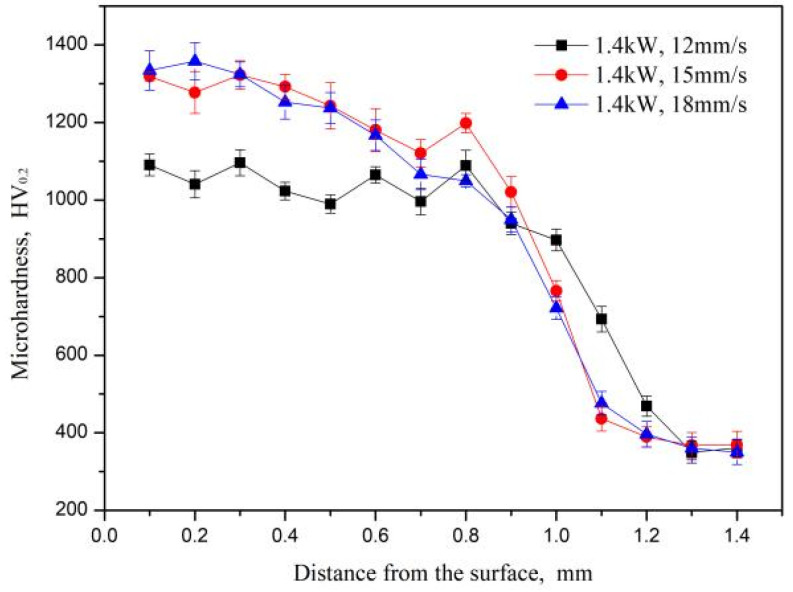
The hardness distributions of the coatings under different scanning speeds.

The microhardness distribution curves of the coatings along the depth direction at different scanning speeds are shown in Figure 7. The microhardness of the samples present obvious differences in the three regions of the substrate, the heat-affected zone and the coating. The overall the microhardness data show a step-like trend and the highest value of microhardness is located near the top of the coatings. In the laser alloying process, the content of carbon atoms in the upper zones of the molten pool is higher, which is beneficial to promoting the in-situ formation of the ceramic-reinforcing phase TiC with a high hardness, resulting in a higher microhardness at the top of the coating. Deeper into the coatings, the dilution effect of the molten substrate, Ti, leads to a significant reduction in the microhardness. In addition, as can be seen from the microstructure analysis, a heat-affected zone exists at the interface of the substrate, possessing slightly higher microhardness than the substrate due to the quenching effect.

For the upper zones of the coatings, the microhardness is improved with the increase of the scanning speed. At a scanning speed of 12 mm/s, the laser beam stays on the surface of the pre-coating for a long time and the molten pool receives more energy. Meanwhile, the cooling rate decreases and the grain size grows larger, resulting in the relatively low microhardness of 1099.4 HV_0_._2_. In contrast, the hardness increases significantly when the laser scanning speed is 15 and 18 mm/s, up to 1320.6 and 1357.7 HV_0_._2_, respectively, which is about four times that of the substrate (340 HV). This is due to the short residence time of the laser beam and the high cooling rate of the molten pool. The shorter formation time of the reinforced phase refines the grain size, and the irregular block structure is reduced, which plays the role in fine-grain strengthening for the high microhardness of the coatings.

The heat-affected zone of the substrate has a hardness between 340 and 400 HV_0.2_ due to the formation of a martensite structure, with a higher hardness when compared to the Ti-6Al-4V substrate. Subsequently, the microhardness of the heat-affected zone also varies with the scanning speed. At a low scanning speed, a large amount of the substrate is melted into the molten pool and a large amount of TiC is generated by this reaction. At the same time, while under the influence of Marangoni convection in the molten pool, TiC is brought to the medium and lower zones of the molten pool to distribute evenly. Therefore, the hardness distribution of the melted zone is the most uniform.

### 3.5. Wear Resistance 

**Figure 8:**
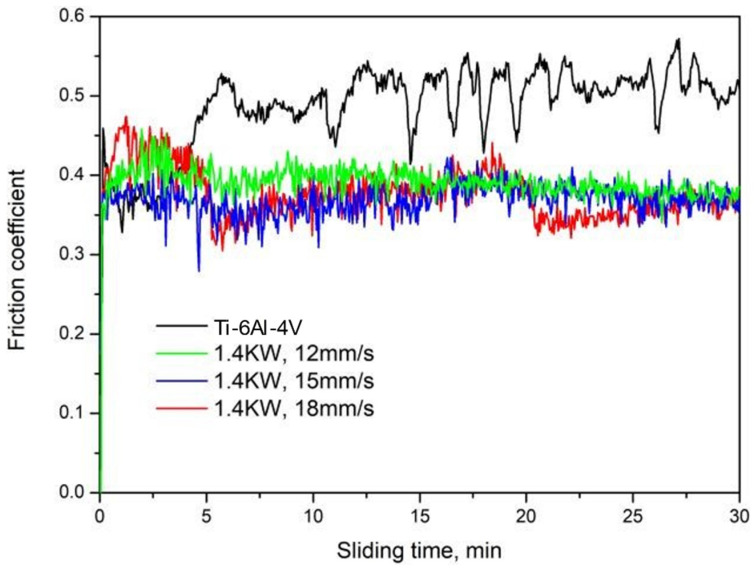
The friction coefficient curves of the substrate and coatings under different scanning speeds.

The friction coefficient curves of the coatings at different scanning speeds are shown in Figure 8. It can be seen from the variation of the curves that the friction coefficient of the coating increases sharply at a beginning of the wear test until a plateau is reached after a short period of time. This is due to the fact that the low level of surface roughness of the specimen in the initial stage of friction guarantees a small coefficient of friction. During the wear test, the roughness of the opposite grinding surfaces gradually increases, and the friction coefficient increases accordingly. After a certain period during the run-in stage, the opposite grinding surfaces gradually enter the stable friction and wear stage. At this time, the average friction coefficient of the substrate is 0.506, while the average friction coefficient of the coatings are 0.389, 0.369 and 0.367 for the scanning speeds of 12, 15 and 18 mm/s, respectively. When the friction coefficient of the coatings is lower than that of the substrate, it indicates good wear resistance. Due to the distribution of a large amount of TiC hard phase in the composite coatings, the adhesion tendency between the composite coating and the friction pair can be effectively reduced, thereby reducing the friction coefficient of the coatings.

**Figure 9:**
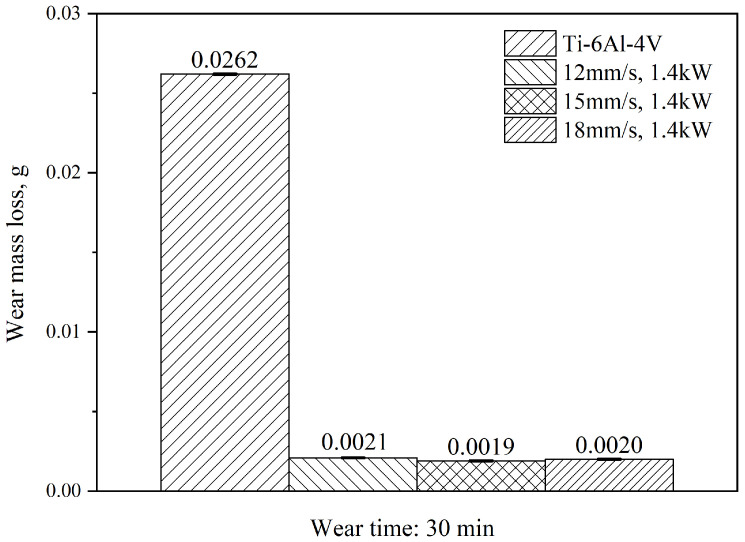
The wear mass loss of the substrate and coatings under different scanning speeds.

The wear loss of the Ti-6Al-4V substrate and the coatings after 30 min of the wear test is shown in Figure 9. The wear loss of the substrate was 0.0262 g. With the increase in the scanning speed, the wear loss of the coating was 0.0021 g, 0.0019 g and 0.0020 g, and this increased to 12.48, 13.78 and 13.10 times that of the substrate, respectively. The wear loss of the coating was significantly smaller than that of the substrate, also indicating significantly improved wear resistance. At the scanning speed of 15 mm/s, the high microhardness and dense structure improved the wear resistance of the coating, with the lowest coefficient of friction and wear weight loss.

**Figure 10:**
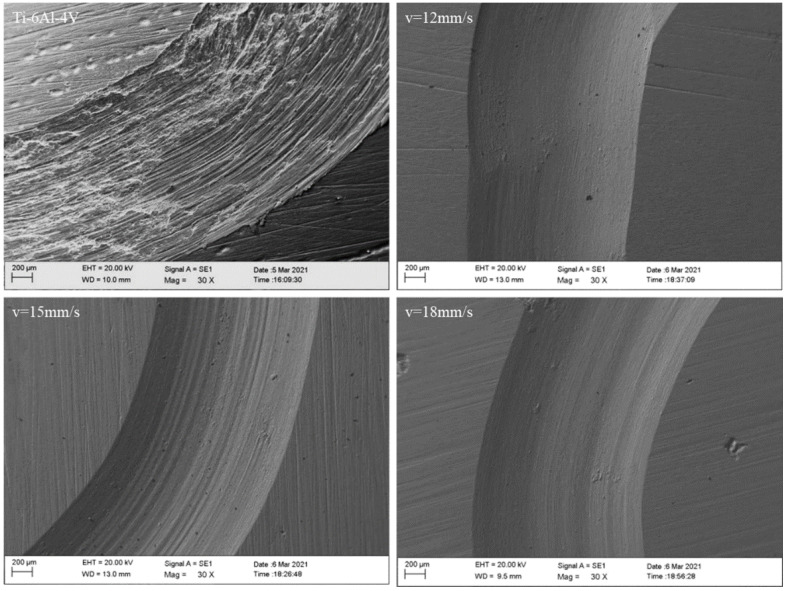
Wear surface morphologies of the substrate and coatings with different laser scan speeds.

**Figure 11:**
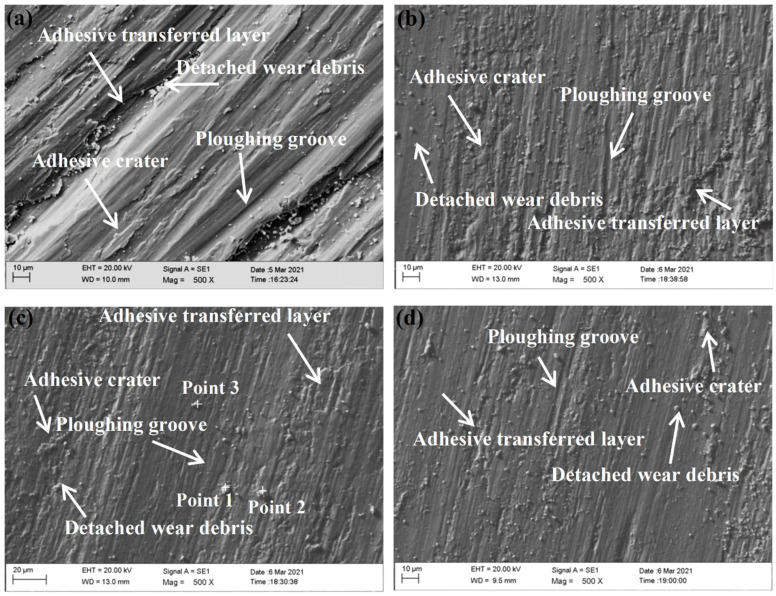
Wear surface morphologies of the substrate and coatings with different laser scanning speeds: (**a**) Ti-6Al-4V substrate, (**b**) 12 mm/s, (**c**) 15 mm/s and (**d**) 18 mm/s.

Figure 10 shows the low-magnification wear morphologies of the substrate as well as the coatings at different scanning speeds. Deep grooves are observed on the surface of the low-hardness substrate, attached with fine wear debris as shown in Figure 11a. The surface grooves of the coatings are shallower, and the wear width is also reduced. The strengthening phases in the coatings effectively resist the indentation of the micro-convex and prevent plastic deformations during the wear test. As a result, a reduced number of peelings occur on the surface of the wear scar. When the laser scanning speed is 15 mm/s, due to the higher microhardness, the furrows on the surface of the wear scar are shallower, as shown in Figure 11c.

**Figure 12:**
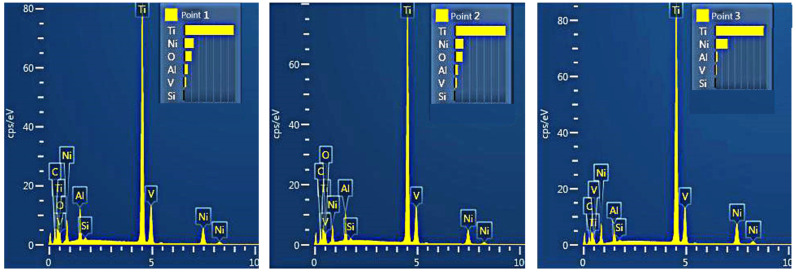
EDS analysis results of wear debris in Figure 11c.

Figure 11 shows the magnified morphologies of the wear–scar surface on the substrate and the coating. Plastic deformations, ploughing, and spalling can be observed on the wear–scar surface of both the substrate and the coatings, indicating that the wear mechanism is the result of the combined effect of adhesive wear and abrasive wear. The grinding balls have a serious grinding effect on the low hardness surface of the substrate under the action of alternating stress, forming deep furrows. The large material transfer resulted in the heavy grinding loss of the substrate (Figure 11a). In contrast, the wear scar surfaces of the coatings are smoother and less worn (Figure 11b–d). This is due to the presence of the TiC hard phase that significantly increases the overall hardness, making it effective against the ploughing action of the grinding balls. When the grinding ball is pressed into the coating, the exposed TiC particles generate torque opposite to the motion of the grinding ball [38], effectively resisting the wear of the coatings. Figure 12 presents the results of the EDS analysis of the wear debris in the coatings (Figure 11c). The results suggest that the wear debris contains a certain amount of O in addition to elements such as Ni and Ti, which is due to the elevated temperature during the friction process, promoting the production of oxides.

## 4. Conclusions

This study employed G@Ni as the alloying material to prepare coatings with high surface quality and metallurgical bonding on a Ti-6Al-4V substrate using laser alloying. The phase composition of the coating mainly consists of TiC, γ-Ni, NiTi and NiTi_2_. Complex reactions happen in the molten pool during the laser alloying process, where Ti reacts with C to form a TiC ceramic-reinforced phase. Ni and Ti react with each other to form various intermetallic compounds such as NiTi and NiTi_2_. Due to the excellent compatibility between the matrix and the reinforced phase generated *in situ*, the hardness and wear resistance of the coating has been improved significantly. Coatings at different scanning speeds have the same composition of the phases while differing in relative content. Under the conditions of 1.4 kW laser power, the increase of the scanning speed refined the microstructure of the coatings. At the scanning speed of 18 mm/s, the coating achieved the highest hardness of 1357.7 HV_0_._2_ and the lowest average friction coefficient of 0.369. At the scanning speed of 15 mm/s, the wear loss of the coating is the smallest and the wear resistance is 13.79 times that of the substrate.

## Figures and Tables

**Table 1:** The chemical composition of Ti-6Al-4V titanium alloy.

Elements	Ti	Al	V	Fe	C	N	H	O
Contents (wt.%)	Balance	6.0	4.1	0.18	0.01	0.01	0.006	0.12

## Data Availability

The data presented in this study are available on request from the corresponding author.
